# Dendritic Cells in Cancer Immunotherapy Clinical Trials: Are We Making Progress?

**DOI:** 10.3389/fimmu.2013.00454

**Published:** 2013-12-13

**Authors:** Lisa H. Butterfield

**Affiliations:** ^1^Departments of Medicine, Surgery and Immunology, University of Pittsburgh Cancer Institute, University of Pittsburgh, Pittsburgh, PA, USA

**Keywords:** cancer vaccines, tumor immunology, antigen presentation, dendritic cells, antigen loading

## Abstract

Dendritic cells (DC) have been tested in cancer immunotherapy clinical trials for two decades. Over this time, the methods of DC culture (or manufacture) have evolved, the approaches for antigen loading have broadened, the maturation signals have varied and different sites of administration have been tested. The post-vaccination immunologic questions asked have also varied between trials and over time. In this review, I will consider multiple aspects of DC-based vaccines tested in cancer patients, including the cell culture, antigen loading, maturation, and delivery, as well as what we have learned from testing immune responses in vaccinated patients who have benefited clinically, and those who have not measurably benefited.

## Introduction

### Beginnings

**Figure 1:**
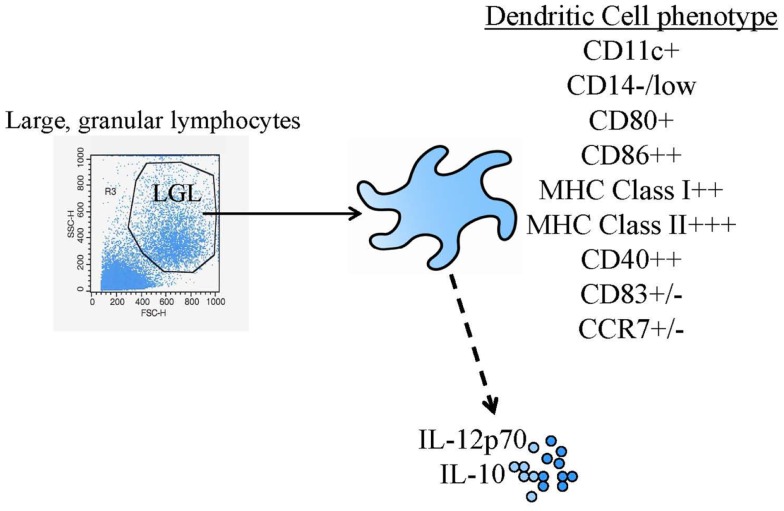
**Common DC vaccine phenotypic and functional assessments**. The diagram shows the most common identity (flow cytometric phenotyping) and potency (cytokine production) tests performed.

Dendritic cells (DC) were first identified in the early 1970s (1). However, the extremely low frequency of these cells in peripheral blood and many tissues made experimentation with DC challenging. General agreement on cell surface markers to uniquely identify “DC” from other myeloid lineage cells was another early hurdle in the field that was surmounted (DC are, at a minimum, large, granular lymphocytes that are MHC class I, MHC class II, and CD86 high, Figure 1). The more widespread investigation of DC identity and biology, and subsequent clinical testing of DC-based vaccines required methods for small and large-scale culture and expansion of DC progenitors *in vitro* (2). Methods were initially identified for expanding DC from human peripheral blood monocytes with granulocyte-macrophage-colony stimulating factor (GM-CSF) (3, 4) and eventually, similar approaches and surface markers were found that could be utilized for both human and murine systems. After these advances, the field was wide open.

In one of the earliest trials, Mukherji et al. (5) used intradermal injection of MHC class I-restricted MAGE-1 peptide-pulsed and GM-CSF-cultured monocytes to treat three HLA-A1^+^ patients with advanced metastatic melanoma. They observed autologous melanoma-reactive and peptide-specific CD8^+^ T cell responses, but no significant therapeutic responses. Such very early clinical results supported the safety and immunologic activity of these cells in cancer patients.

### First generation clinical trials

The early clinical trials of DC-based cancer immunotherapy established the general safety and feasibility of this cancer vaccine strategy, and its lack of toxicity compared with other cancer treatment approaches (e.g., chemotherapy, radiation). Importantly, a small number of positive clinical responses and the clear demonstration that the goal of anti-tumor immune activation was achieved, bolstered the field, and supported additional trials. There have been several recent DC vaccine reviews published that are excellent, and that give additional details (6–8). The few early trials highlighted below are important, but small, and did not utilize standardized manufacture procedures throughout the clinical trial.

One of the first reported clinical trials that described the ability of tumor antigen-pulsed DCs to elicit a tumor-specific T cell response and yield a clinical response was published by Hsu et al. (9). In this study, four patients were treated with low-grade follicular B-cell lymphoma resistant to chemotherapy. The DCs were pulsed with target antigens of clonal immunoglobulin (idiotype) expressed by the non-Hodgkin’s lymphoma, a tumor-specific, unique antigen. Patients were immunized with DC followed by booster injections of idiotype protein and keyhole limpet hemocyanin (KLH, as an immunogenic xenoantigen as well as heterologous “help” to activate CD4^+^ T cells) as well as a final DC boost infusion given 5–6 months later. All four patients developed cellular proliferative responses specific to their own idiotype protein. More importantly, one patient had a complete tumor regression, a second patient had a partial regression, and a third patient resolved all evidence of disease. This very small study was an important proof of principal for the clinical potential of DC vaccines.

While the study performed by Mukherji et al. (above) evaluated monocyte-derived antigen presenting cells (APC), it may not have formally tested a more fully differentiated DC because the culture contained GM-CSF, but it lacked IL-4. The first clinical trial using the monocyte-derived DC that have been most commonly used in clinical trials (including both GM-CSF and IL-4 in the monocyte precursor culture) was performed by Nestle et al. (10). Sixteen melanoma patients were treated using autologous monocyte-derived DC pulsed with a cocktail of gp100, MART-1, tyrosinase, MAGE-1, or MAGE-3 peptides chosen to suit the individual patient’s class I HLA molecules. In addition, DC pulsed with autologous tumor lysate were used to treat another four patients. To provide antigen non-specific CD4^+^ T cell-mediated help for the CD8^+^ T cells, KLH was included during antigen pulsing. DC were injected directly into uninvolved lymph nodes. Patients received 6–10 injections of 1 × 10^6^ cells every 1–4 weeks. Tumor regression was seen in 5 of the 16 patients, including two complete responses lasting over 15 months. Tumor regressions occurred in skin, soft tissue, lung, and pancreas indicating an impact on the clinical course of metastasizing melanoma, regardless of metastatic site.

As with many of the early trials (examples here and others), a variable number of DC vaccine administrations, consisting of different cell numbers and boost injections were delivered, and multiple types of antigen loading strategies were used. These earliest clinical studies were more proof of principle for the *in vivo* activity of DC, and less a formal testing of a specific DC vaccine approach.

In another melanoma clinical trial, Banchereau et al. (11) evaluated immune and clinical responses in 18 patients with metastatic melanoma after injecting DCs pulsed with peptides (MART-1, tyrosinase, MAGE-A3, and gp100) subcutaneously. They utilized CD34^+^ hematopoietic progenitor cells as an alternative source of DC. DC were administered in a dose-escalation design. Enhanced antigen-specific immune responses to at least one of the peptides were seen in 16 or 18 patients, and 6 of 7 patients with immunity to two or fewer antigens had progressive disease after the study ended, while only 1 of 10 patients who responded to more than two antigens had tumor progression. This larger and more standardized study showed that broad immune responses to multiple tumor antigen-derived peptides correlated with better clinical outcome, one of the first studies showing that statistically significant correlation between immunity induced from DC vaccines and clinical outcome.

**Figure 2:**
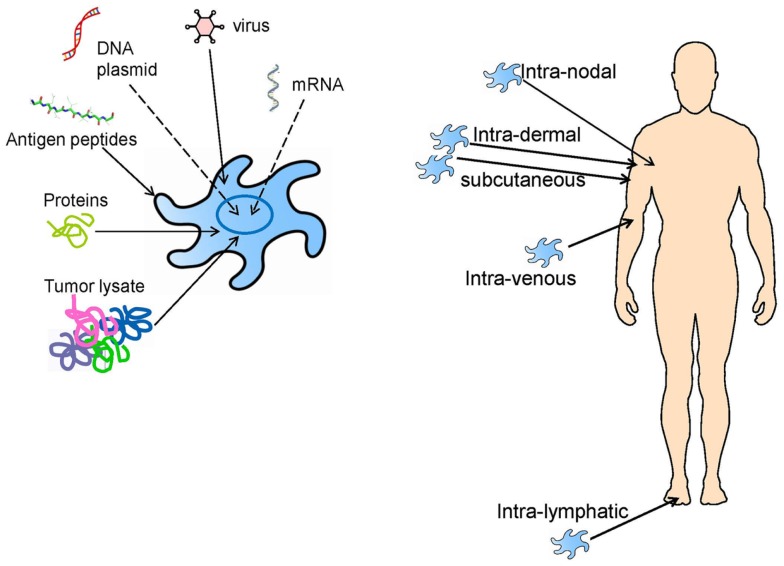
**DC vaccine loading and administration approaches that have previously been tested in clinical trials, and which are still being investigated today**.

It is clear from the clinical trials described above, that most clinical trials are unique, they involve individual patient vaccination approaches and single clinical trial arms, and it is difficult to compare them. Monocytes and CD34^+^ progenitors; complex tumor lysates containing normal, tumor-associated/shared and tumor-specific/private antigens, or synthetic MHC class I-restricted peptides; injection into blood (i.v.), skin (s.c. or i.d.), or lymph nodes (i.n.); are all parameters playing unclear roles in any clinical responses seen (Figure 2). The initial lessons learned were simply that DC-based vaccines were safe, feasible, and had the potential to promote clinically significant tumor regressions.

### Limited clinical results for DC-based vaccines

In 2004, Rosenberg et al. published an article on the state of active specific immunotherapy cancer trials (12). They analyzed 9 years of data (1995–2004), essentially all of the early, or “first generation” trials. Overall, they reviewed 1,306 solid tumor patients using the modified Response Evaluation Criteria in Solid Tumors (RECIST) in which clinical response is defined as at least 50% reduction in the sum of the products of the perpendicular diameters of all lesions without 25% growth of any lesion or the appearance of new lesions. With an overall therapy-induced tumor regression rate of only 3.3% in patients vaccinated with synthetic peptides, “naked” DNA, peptide-pulsed DC, recombinant vaccinia viruses, recombinant fowlpox viruses, or recombinant adenoviruses expressing various tumor-associated antigen (TAA), the results were grim for vaccine approaches in general. Of these immunization methods, peptide-pulsed DCs seemed to be the most effective strategy, with 7.1% of treated patients exhibiting tumor regression. While this frequency of response was higher than those frequencies found for other vaccination strategies, the clinical response was still low.

### DC vaccine complexities

Unlike chemotherapy, immunological vaccines have not followed a linear dose-response effect. Instead, because immunologic vaccines depend on the complex interactions of a large number of variables, many of which are difficult to test: (1) the administration route (s.c., i.d., i.v., i.n., and more recently, intra-lymphatic, i.l.), (2) minimum immunogenic dose, (3) higher dose effects, (4) vaccination schedule (weekly, monthly, or multiple times in a week or month), (5) immunological adjuvant type, and (6) the existing state of host immunological competence. There have been attempts to make “immune competence” a criterion for trial enrollment. A standardized skin test to recall antigens like tetanus and mumps was investigated, but the results were unrelated to vaccine immune response. Better measures of “immune health” are under investigation (13) but there are no clear definitions to date that might serve to identify patients who are most likely to benefit. Any alterations of these many variables can impact the patient immunologic as well as clinical outcome following therapeutic immunization.

The majority of patients treated in these earlier studies were late-stage metastatic patients that were heavily pretreated with conventional chemotherapeutic drugs prior to immunizations. Not only do late-stage tumors have potent immune-inhibitory functions well established both in the tumor microenvironment and systemically, but many traditional chemotherapies have also been shown to non-specifically decrease the number of leukocytes in recipients, making metastatic patients severely immune-compromised. Such patients would also be expected to have multiple tumor resistance mechanisms in place (e.g., infiltrated regulatory T cells, myeloid-derived suppressor cells, and other immature and skewed macrophages, immuno-inhibitory cytokines, and genetic heterogeneity in tumor subclones). Assessment of tumor infiltration and inflammation is being investigated as a biomarker for responsiveness to not only vaccines, but also other immunotherapy approaches and traditional cancer treatments (14, 15), but these areas of investigation are relatively recent and still being validated.

There are several other possibilities to explain the poor clinical response to these vaccines. The immune system, while potentially effective, is limited by the frequency of responders that can be stimulated by vaccinations. Even if TAA-specific responses were stimulated by immunization, it is possible that the bulk tumor mass was too large at the time of treatment for the available effector T cell population to infiltrate it and eliminate it efficiently. It is also possible that while the vaccine-targeted antigens are expressed by the tumors, their derivative peptides are not presenting on the cell surface in the context of MHC class I molecules, making the tumor cells effectively invisible to CD8^+^ T cell recognition. Tumors can down-regulate antigen processing machinery molecules, including β-2-microglobulin (16, 17). Another possibility is that TAA used for vaccinations were not expressed by targeted tumors because metastatic deposits do not necessarily express the same repertoire of antigens as the primary tumor or that TAA-derived peptides used were not effective at eliciting high-avidity T cell responders. This heterogeneity has been observed in melanoma (18). The highest avidity T cells specific for self antigens may have been deleted during the development of the immune system by normal negative selection. Therefore, for some patients treated in early DC vaccine trials, instead of receiving a vaccine tailored to the individual patient’s TAA repertoire, these individuals may have been treated with arguably irrelevant non-presented or weakly immunogenic antigens that led to a clinically meaningless immune response. Vaccines targeting only CD8^+^ T cells, with short MHC class I-restricted peptides, may have only been able to activate “helpless” CD8^+^ T cells with functional defects (19). Lastly, some tumors have evolved cell-autonomous resistance to immune-mediated killing.

### Sources of tumor antigen: Immune target complexities

Tumors are not homogenous tissues that can be effectively treated with a single antigen epitope vaccination tactic. Tumors vary in physiological location (primary tumor sites and metastatic sites), TAA repertoire, vascularization, surrounding stroma, and other properties. Some tumor types are considered more “immunogenic” due to spontaneous immune infiltration and have, therefore, been an early focus for many DC-based immunotherapy trials (melanoma, renal cell cancer). These variations in tumor biology, immune infiltration, and microenvironment biology are observed between patients, the tissues affected, and at different time points in the malignant process. For example, when considering inclusion criteria, “stage IV cancer patients” are not a homogeneous group. Whether patients with brain metastases can be included, or those with LDH levels above normal limits must be considered, as immunotherapy vaccine clinical responses can need time to evolve, and not all clinical settings are expected to allow for immune response evolution.

When considering autologous tumor-based immunization strategies, there are types of cancer that are not generally surgically removed (pancreatic cancer, hepatocellular cancer treated with ablative techniques), so the ability to load DC with autologous tumor as a source of all potential public and private TAA may not be feasible. Established cell lines are an immortal source of antigen, but may have limited antigenic overlap with a specific patient’s tumor. Cell lines may express a few known, shared TAAs, however they may not express any tumor-specific and/or mutated/private antigens that the patient’s tumor expresses and which may be critical to clinical outcomes. Similarly, some tumor types may have only a few characterized shared TAA with even fewer well-defined HLA-matched peptide epitopes. Importantly, since the expression of TAA is not uniform among tumor cells and metastases, it may be critical to co-administer several antigens, rather than a single one, to avoid the possibility that the sole TAA will prove non-immunogenic or that its epitopes may not be adequately presented on the tumor cell. A long term goal in the field has been to identify the “best” TAA for targeting with vaccines. The optimal TAA would be critical to survival of the tumor cells, expressed at distant metastatic sites (not downregulatable), specific to the tumor (not expressed on normal tissues), and immunogenic. Characterized TAA were ranked by a group of experts (20), but the ideal, defined, shared targets have not been identified for many tumor types, and there remains some disagreement in the field exactly what type of antigen should be targeted.

## Technical Issues and Remaining Questions Regarding DC Vaccines

### Maturation

An early lesson learned in DC vaccine development was that the DC obtained after 5–7 days of culture with GM-CSF+IL-4 were not in an optimal state for T cell activation. These DC were subsequently referred to as “immature” and potentially tolerogenic until triggered by a pathogen and/or inflammatory signal. Such signals serve to upregulate antigen presentation and co-stimulation molecules and function, and reduce antigen uptake. Early cocktails were sometimes donor-specific and undefined (monocyte-conditioned medium), weakly stimulatory (TNF), or contained molecules which were subsequently shown to have some negative effects (PGE_2_). Currently employed cocktails can incorporate specific pathogen-derived molecules, toll-like-receptor ligands (TLRs) and other “type 1” skewing agents including interferons (21). Conversely for clinical settings other than cancer, immune suppressive cocktails can be used to push DC toward their tolerizing capabilities, for autoimmune or transplantation settings. For example, anti-sense oligo co-culture of DC with anti-sense CD80, CD86, and CD40 for treatment of autoimmune diabetes has been tested, or DC culture with vitamin A for inhibition of transplant-specific immunity is being developed (22, 23). Overall, as environmental sensors, DC can be significantly modulated by instructions delivered by maturation signals and optimal signals for DC vaccines are still being developed.

### Dose and route

Many new therapeutic drugs are tested for the effective, maximum tolerated, and toxic doses in early clinical trials. Their routes of administration are often intravenous for quick dissemination to many anatomic sites. DC vaccine development has not yet shown significant toxicity at any dose delivered (24, 25), and there have been few suggestions of minimum required dose. Doses are largely defined as the “maximally feasible dose” from a blood draw or leukapheresis procedure of a specific duration (90 min to 4 h). Regarding route of delivery, many options have been tested and questions remain, each has positive and negative aspects to consider. For intradermal delivery, too many DC in a small volume might die *in situ*. Intra-nodal delivery may deliver the DC to an optimal site, unless they are not injected into a cellular region and are injected in fat or stroma instead (26). Intravenous delivery may send cells to lungs and liver and not secondary lymphoid tissues. Not all tumors are accessible for intra-tumoral injection (which has been tested with unloaded DC to allow DC to directly sample TAA), and that environment might be harsh and result in quick loss of DC function or viability *in vivo*. Intra-lymphatic delivery may also be immunologically ideal (like intra-nodal), but is clinically challenging to administer. The optimal DC vaccine dose and route also remains to be established for human clinical trials.

### Second generation trials and lessons learned

A new generation of clinical trials was conducted from 2004 to 2012, testing new hypotheses based on the lessons learned from the first generation, proof-of-principle studies. One key area of change has been the use of defined, optimized cytokine cocktails and pathogen-derived agonists to mature DC. The individual constituents of these cocktails have an important impact on DC biology, including the relative level of cell surface molecules (e.g., co-stimulatory molecules CD80, CD86, or maturation markers CD83 or CCR7), the amount, timing, and duration of cytokine production by DC (e.g., IL-12p70, IL-12p40, IL-10), DC lifespan, and the trafficking potential and response to chemokine gradients (21). Early, high level production of IL-12p70 may not be as optimal for T cell activation *in vivo* as delayed IL-12 production until after DC have arrived from the site of injection to the lymph node. Newer DC vaccines are not simply “mature” by a few phenotypic markers, but are treated to elicit specific types of “maturity” or immunologic skewing, based on culture conditions, and planned antigen loading and injection route strategies. Some trials testing more optimal cocktails have been performed and published results should be available soon. Other cytokine culture conditions have been tested *in vitro* [with IL-15 or IL-13 (7, 8)] but access to clinical grade reagents has been a limitation until more relaxed guidelines from the US FDA, at least for the earliest stage trials (“Guidance for Industry: CGMP for Phase I Investigational Drugs” http://www.fda.gov/downloads/Drugs/GuidanceComplianceRegulatoryInformation/Guidances/ucm070273.pdf).

Shorter duration of DC cell culture (2–3 days instead of 5–7 days with different DC maturation triggers has also been tested (27). It is difficult to identify superior DC *in vitro* given that DC differentiation may not be fixed once the DC are administered, and very few randomized trials have been performed which compare different DC *in vivo*. This also leads to one of the major limitations in DC trials. The size of most trials is quite small and hence, there are not multiple trial arms to compare experimental groups and learn the answers to important questions (with any statistical confidence). Even when trials are “negative,” showing minimal positive clinical effects, there are still many variables which might explain weak results, including unavoidable patient–patient variation in generation of an autologous cellular product, and insufficient funding available to run larger, randomized trials testing specific variables, like maturation cocktails, antigen choice, antigen loading, dose, and route (28).

A blow to the field was the result of the randomized Phase III trial comparing DTIC chemotherapy for melanoma (which has a very poor efficacy record) with a matured, peptide-pulsed DC vaccine (29). The trial was stopped early due to lack of differences between the trial arms, which had similar overall clinical response rates of <6%. Many of the variables discussed above have been hypothesized to have played a part in the disappointing DC vaccine results, including DC vaccine quality and consistency in manufacture between different manufacturing sites, site of delivery (s.c. instead of i.d.), and lack of tumor-specific helper epitopes or heterologous help to promote CD4^+^ T cell activation. With so many open questions on how best to prepare DC vaccines, there are still many possible causes for minimal clinical responses.

However, there have been a number of more successful trials published in this period. Objective clinical responses and significant immunologic responses were observed in a trial in renal cell carcinoma patients testing Muc-1 peptide+heterologous PADRE pulsed DC (s.c.) (30). These DC vaccines were combined with low dose IL-2. In another study, acute myeloid leukemia patients in remission from previous standard therapy receiving WT-1 mRNA-loaded DC vaccines showed immune activation and improved clinical outcomes (31). Another trial tested DC-tumor fusions in myeloma patients before or after autologous stem cell transplant, and observed both anti-tumor immune activation and reduction of disease (32). Interestingly, these trials all employed a DC vaccine combination strategy.

## Next Generation Trials: Where are We Going?

### DC for cancer prevention?

A new clinical setting for tumor antigen vaccination has been proposed, focused on prevention of cancer development in high risk patients without current disease (33). This vaccination setting was recently reported for a Muc-1 peptide-based vaccine in subjects with advanced colonic adenomas (but not yet colorectal cancer). The results showed that the peptide vaccine was immunogenic in 43% of the subjects and response was inversely correlated with circulating MDSC levels (34). A related Muc-1 vaccine improved survival in a murine model of colitis-associated colorectal cancer (35), supporting the further testing of preventative cancer vaccination, including utilizing DC, in a prevention setting.

### Antigen loading

For antigen loading, peptide-pulsing, transfection/transduction, and protein-pulsing continue to be used, as well as tumor lysate loading. The procedure followed for tumor lysate preparation has recently been examined. The simplest approach has been multiple rounds of freezing (in a dry ice/ethanol bath or at −80°C) and thawing (by 37° water bath). This procedure can break open cells in a manner mimicking necrosis and allow subsequent tumor protein isolation. However, there are other approaches. Tumor cell exposure to UV and gamma irradiation has been shown to mimic apoptosis, which delivers different signals to DC than necrotic cells. Recently, tumor treatment with hypochlorous acid before lysate purification was tested (36), and this method of oxidation and rapid necrosis may be superior for DC vaccine loading. Another important element may be the changes in tumor antigen expression when tumors are cultured in hypoxic conditions (5% instead of 20%) specifically mimicking *in situ* hypoxic tumor oxygen levels (37). Such improved antigen preparation approaches may yield improved clinical outcomes.

### Route of delivery

Based on data demonstrating that DC vaccines delivered intradermally (i.d.) show very low level (<2%) migration to lymph nodes (based most often on ^111^In-labeling (38, 39), and that ultrasound-guided intra-nodal delivery has a risk of the vaccine being injected into fat instead of a cellular area (26), other routes of delivery have been tested. The results have varied between mice and humans, and in patients, and all tested routes of delivery have proven to be immunogenic in terms of T cell response induction. Without higher rates of objective clinical responses, identification of superior routes of delivery remain unknown. Thus far, there has also been no strict correlation between phenotypic measures, like CCR7 level on the DC surface (40), and subsequent migration. There are suggestions that the maturation cocktail used impacts migration (40) but there are no definitive answers yet. More recently, newer MRI-based DC vaccine labels have been tested (41) (D. Bartlett and P. Kalinski, personal communication, 2013) and prolonged, semi-continuous intra-lymphatic delivery of DC has been tested [(42), and P. Kalinski, personal communication, 2013]. Continued efforts at tracking DC migration *in vivo* and optimizing routes of delivery may yield more potent DC vaccines. A few such DC trials are underway.

### Suppressive DC

While the cancer and infectious disease communities have investigated optimally immune stimulating DC, the organ transplantation and autoimmunity fields have sought approaches to either maintain an “immature” DC status, or differentiate DC toward a tolerogenic or immune suppressive activity. Such strategies include pulsing DC with anti-sense oligonucleotides for co-stimulatory molecules CD40, CD80, and CD86 to downregulate these molecules (22) for prevention and treatment of diabetes, or culture with vitamin D3 and IL-10 for allograft tolerance (23). A recent Phase I clinical trial testing anti-sense CD40/80/86 oligo pulsed DC in type 1 diabetic patients showed that the cells were safe, well-tolerated, and resulted in a reduction in a subset of B cells (22). In recent preclinical primate modeling of kidney allograft survival, i.v. infusion of vitamin D3/IL-10 regulatory DC was also safe and resulted in significantly improved allograft survival (23). Further clinical development of these strategies are underway.

### DC vaccine combinations

The field of cancer immunotherapy is now in the position of having more effective drugs encompassing not only vaccines in development, but small molecule inhibitors of key signal transduction pathways and immunologic checkpoint inhibiting antibodies. While all of these modalities, like the traditional standards of care (surgery, chemotherapy, radiation) have strengths and weaknesses, the current generation of clinical trials focuses on combinations of these approaches. DC vaccines may have limitations as stand-alone therapeutics, but in combinations they could play a role in initiating and boosting anti-tumor immunity, promoting *in vivo* cross-presentation, and promoting long term immunologic memory. Cytotoxic treatments can have multiple positive effects on the immune system, from simple release of tumor antigens as cancer cells die, to cytotoxic agent-specific effects. Release of tumor antigens allows endogenous DC to take up and present them, or for larger numbers of tumor bed injected DC to take up the broad array of released tumor antigens for T cell activation. Cytotoxic agent-specific immune effects can include: upregulation of immune stimulatory molecule expression on tumor cells (e.g., DAMPs), increased tumor antigen expression, reduced suppressor cells frequencies, as well as increased CTL proliferation and activation. The pioneering studies in this area have largely been performed in murine models, but the immune-promoting effects of non-immune-based therapeutics are now being assessed in clinical trials. Future DC vaccine combinations with rationally chosen agents may increase the effectiveness of DC vaccines (43, 44).

### DC transcriptome analysis

An important technological breakthrough has been the ability to test the DC vaccine transcriptome. This detailed molecular characterization allows for a broader understanding of DC vaccines. Manufacturing conditions, different maturation cocktails (45, 46), and their impact on DC biology, over and above even a very thorough examination of DC surface phenotype and cytokine production (47) can be examined on a molecular basis. To date, surface expression of standard DC markers (CD80, CD83, CD86, MHC class I, MHC class II, CCR7) has not correlated significantly with *in vivo* vaccine effects. The type-1-skewing cytokine produced by DC, IL-12p70, is actively being investigated as a potency assessment, based on its significant correlation with clinical outcome was demonstrated (48). This assay was employed after it was standardized for both spontaneous and induced expression of IL-12p70 heterodimers (47). Transcriptome analysis allows for a much broader assessment of DC vaccines, and may prove informative for predictive biomarkers of immune and/or clinical response. Such profiling has identified a type-1-skewing genetic profile expressed by DC matured with IFNγ+LPS (45), and a list of candidate genes that may be helpful for identity, stability, and potency measures of DC vaccines (46). This approach may also identify patient-to-patient variation of immunologic significance.

### Immunologic monitoring

Each DC vaccine clinical trial is based on the hypothesis that optimal tumor antigen presentation will promote clinically effective anti-tumor immunity. Understanding the effects of the vaccines on each patient’s immune system is of utmost importance in moving the field forward. Most trials examine effector T cells activated by antigen-pulsed DC, but the cross-talk between DC and innate immune cells may also be mechanistically very important. Vaccine cell interaction with innate immune cells is expected to be variable with different types of DC cultured and loaded in different ways. DC modulation of suppressor cells, like regulatory T cells and myeloid-derived suppressor cells, may occur, and the overall immune effects may vary in magnitude and quality between peripheral blood and the tumor site. Some studies have found similar results in blood and tumor, while others have not, and studies examining DC vaccine effects at the tumor site are limited. Obtaining tumor biopsies can be challenging but well is worth the difficulties in order to understand the direct tumor site impact. Larger sized DC trials may involve multiple clinical sites, as well as vaccine manufacture sites, which necessitates careful standardization of blood processing vaccine culture and immune monitoring assay methodologies (49) as well as data reporting (50). Despite the technical challenges, careful immunologic monitoring, particularly with multiple functional assays, yields critical mechanistic insights.

## Conclusion

Yes, we are making progress in the DC vaccine field. A more rational, defined, and data-driven approach is being employed in culturing, maturing, and antigen loading of DC (51). Fully characterizing DC vaccine transcription profiles moves far beyond the limited cell surface phenotypes previously employed. Performing more standardized trial designs where patients receive the same type of vaccine reduces variables to patient-to-patient variation instead of adding variables and may identify the most critical vaccine parameters to carry forward. More thorough, robust and standardized immune monitoring assessments are allowing to field to draw more meaningful conclusions from each trial. In the future, the next generation of optimized vaccines identified may selectively be used for individual patients, based on their tumor biology. A new generation of DC vaccine trials are underway (52) which have the potential to move this area of personalized medicine forward.

## Conflict of Interest Statement

The author declares that the research was conducted in the absence of any commercial or financial relationships that could be construed as a potential conflict of interest. The author and editor declare that while the author Lisa Butterfield and the editor Penelope Morel are currently employed by the University of Pittsburgh, there has been no conflict of interest during the review and handling of this manuscript.
